# Extremely preterm children and relationships of minor neurodevelopmental impairments at 6 years

**DOI:** 10.3389/fpsyg.2022.996472

**Published:** 2022-12-01

**Authors:** Ulla Niutanen, Piia Lönnberg, Elina Wolford, Marjo Metsäranta, Aulikki Lano

**Affiliations:** ^1^Department of Child Neurology, New Children’s Hospital, Pediatric Research Center, University of Helsinki and Helsinki University Hospital, Helsinki, Finland; ^2^Department of Psychology and Logopedics, University of Helsinki, Helsinki, Finland; ^3^Department of Pediatrics, New Children’s Hospital, Pediatric Research Center, University of Helsinki and Helsinki University Hospital, Helsinki, Finland

**Keywords:** extremely preterm, minor neurodevelopmental impairments, motor coordination, multimorbidity, relationship maps, sensorimotor processing, visual-motor performance

## Abstract

**Aim:**

This study investigated minor impairments in neurological, sensorimotor, and neuropsychological functioning in extremely preterm-born (EPT) children compared to term-born children. The aim was to explore the most affected domains and to visualize their co-occurrences in relationship maps.

**Methods:**

A prospective cohort of 56 EPT children (35 boys) and 37 term-born controls (19 boys) were assessed at a median age of 6 years 7 months with Touwen Neurological Examination, Movement Assessment Battery for Children, 2nd edition (MABC-2), Sensory Integration and Praxis Test (SIPT), and a Developmental Neuropsychological Assessment, 2nd edition (NEPSY-II). Altogether 20 test domains were used to illustrate the frequency of impaired test performances with a bar chart profile and to construct relationship maps of co-occurring impairments.

**Results:**

The EPT children were more likely to perform inferiorly compared to the term-born controls across all assessments, with a wider variance and more co-occurring impairments. When aggregating all impaired test domains, 45% of the EPT children had more impaired domains than any term-born child (more than five domains, *p* < 0.001). Relationship maps showed that minor neurological dysfunction (MND), NEPSY-II design copying, and SIPT finger identification constituted the most prominent relationship of co-occurring impairments in both groups. However, it was ten times more likely in the EPT group. Another relationship of co-occurring MND, impairment in NEPSY-II design copying, and NEPSY-II imitation of hand positions was present in the EPT group only.

**Interpretation:**

Multiple minor impairments accumulate among EPT children at six years, suggesting that EPT children and their families may need support and timely multi-professional interventions throughout infancy and childhood.

## Introduction

Studies of preterm-born children suggest a wide range of minor impairments indicating delays or performance difficulties in neurological (Broström et al., 2018; Tommiska et al., 2020), sensorimotor (De Kieviet et al., 2009; Williams et al., 2010; Broström et al., 2018; Spittle et al., 2018; Bolk et al., 2018a; Niutanen et al., 2020), cognitive (Allotey et al., 2018; Twilhaar et al., 2018), neuropsychological functions (Marlow et al., 2007; Geldof et al., 2012; Koivisto et al., 2015; Allotey et al., 2018; O’Reilly et al., 2020), and behavior (Farooqi et al., 2007; Allotey et al., 2018; Broström et al., 2018). Compared to term-born children, preterm-born children score lower in global cognitive tests (Twilhaar et al., 2018), executive (Allotey et al., 2018; Brydges et al., 2018), visual–spatial (Geldof et al., 2012; O’Reilly et al., 2020), and motor functions (De Kieviet et al., 2009; Bolk et al., 2018a). Motor impairments have been documented in the quality of gross-and fine-motor coordination and balance (Hadders-Algra, 2010; Broström et al., 2018) and the quantity of manual dexterity, speed, and balance (De Kieviet et al., 2009; Van Hus et al., 2014; Spittle et al., 2018; Bolk et al., 2018a). Moreover, visual-motor and sensorimotor integration impairments are common in preterm children (Luoma et al., 1998; Marlow et al., 2007; Goyen et al., 2011; Geldof et al., 2012; Lönnberg et al., 2018; Bolk et al., 2018b; Niutanen et al., 2020). Motor impairments frequently co-occur with minor neurological (Hadders-Algra, 2010; Van Hus et al., 2014; Broström et al., 2018), cognitive, learning, and behavioral impairments (Van Hus et al., 2014; Allotey et al., 2018). The impairments tend to become more evident toward school age (De Kieviet et al., 2009; Geldof et al., 2012; Broström et al., 2018; Niutanen et al., 2020) and persist (Poole et al., 2015) or increase over time (De Kieviet et al., 2009; Allotey et al., 2018; Spittle et al., 2018; O’Reilly et al., 2020; Doyle et al., 2021), and complicate daily activities, learning, and participation (Bolk et al., 2018a; Doyle et al., 2021).

According to the literature, the severity of impairments in preterm-born children increases with decreasing gestational age and birth weight (Larroque et al., 2008; Blencowe et al., 2013; Serenius et al., 2016). Studies indicate that 17–30% of EPT children exhibit an overall mild cognitive impairment (Larroque et al., 2008; Johnson et al., 2009; Serenius et al., 2016), 30–52% show mild motor impairments (Goyen et al., 2011; Bolk et al., 2018a; Tommiska et al., 2020), and 39–66% have special educational needs. (Saigal et al., 2003; Johnson et al., 2009, 2016; Tommiska et al., 2020).

Few studies on EPT children provide knowledge on multiple co-occurring minor impairments, including motor performance. It has been suggested that one-third of four-year-old EPT children exhibit more than one neurodevelopmental impairment in motor, cognitive, language, and behavioral domains (Woodward et al., 2009). When comparing EPT children with and without motor impairments, those with motor impairments have more often co-occurring social, behavioral, and attentional impairments (Bolk et al., 2018a) and minor neurological dysfunctions (Broström et al., 2018). Motor, perceptual-motor, and visual–spatial impairments have further been associated with additional co-occurring cognitive impairments (Marlow et al., 2007). However, it is unknown to what extent the impairments are related or vary in extremely preterm children.

We have previously reported in our EPT cohort a positive relationship between newborn neurobehavioral characteristics of visual fixation with sustained alertness contributing to better visual-motor performance at 2 years (Stjerna et al., 2015) and better sensorimotor function at 6 years of age (Aho et al., 2021). We have also shown these EPT children to perform worse than their term-born peers in visual-motor, somatosensory, and motor planning functions at early school age (Lönnberg et al., 2018). Furthermore, we have reported on the importance of maternal sensitivity and supportiveness to positive neurocognitive development in our cohort of EPT children (Rahkonen et al., 2014). In the present study of the same cohort, we aimed to investigate neurodevelopmental outcomes at 6 years of age, focusing on minor neurological, sensorimotor, and neuropsychological impairments by comparing EPT children with term-born controls. We further aimed to identify the most affected domains and their co-occurrences by employing relationship maps to better visualize the extent and relations of the impairments. We hypothesized that multiple neurodevelopmental impairments would be more common in EPT children than in the term-born controls.

## Materials and methods

### Participants

**Figure 1:**
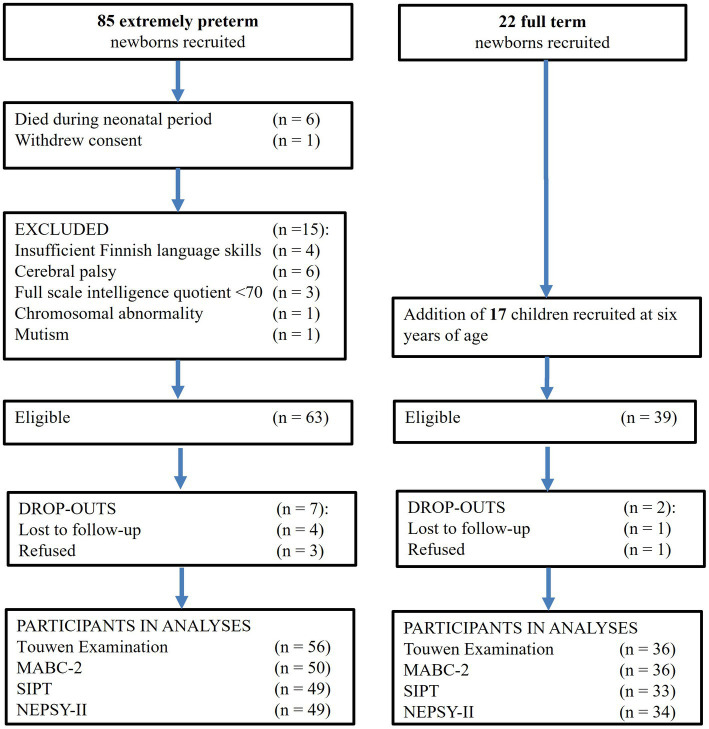
Flow chart of participants in the cohort.

The current study included 56 EPT (<28 weeks of gestation) and 37 term-born control children, originally recruited for a large prospective multimethodological study and followed up since birth (Kekeke-study—Extremely Preterm Birth and Development of the Central Nervous System, see Figure 1, Flow chart of participation). The EPT infants were born between May 2006 and September 2008 and treated after birth at the neonatal intensive care unit of the Helsinki University Hospital in Finland. Six of the infants died during the neonatal period. Children with cerebral palsy, intelligence quotient below 70, blindness or deafness, chromosomal abnormality, mutism, and families with no Finnish language skills were excluded. A control group of healthy full-term infants, born between September 2006 and June 2009, was recruited at birth at the maternity wards of the Helsinki and Uusimaa Hospital District, Finland (*n* = 22) and six years of age by advertising at nursery schools in the Helsinki area and a parents’ association for prematurely born babies (*n* = 17). Inclusion criteria were gestational age from 37 to 42 weeks, birth weight >2,500 g, and no need for monitoring or treatment at the children’s ward after birth.

The Ethics Committee for gynecology and obstetrics, pediatrics, and psychiatry of the Hospital District of Helsinki and Uusimaa approved the study protocol. Participant children and their parents signed informed consent in accordance with the Declaration of Helsinki.

### Clinical data

Demographic data (Table 1) were gathered from parental questionnaires and hospital records. The data encompassed socio-economic status and neonatal morbidity factors for prematurity (Blencowe et al., 2013): bronchopulmonary dysplasia at 36 gestational weeks, the highest grade of intraventricular hemorrhage in serial ultrasound images during the neonatal period, a four-category classification of white matter injury (Woodward et al., 2006) at term equivalent age in brain magnetic resonance imaging, and birthweight below -2 standard deviation (SD) for infants who were small for gestational age according to Finnish growth reference data (Sankilampi et al., 2013). Visual and hearing information was gathered from the child’s health records.

**Table 1:** Characteristics of the extremely preterm and term-born participants and extremely preterm-born dropouts.

	Extremely preterm-born *n* = 56	Term-born *n* = 37	*p*^1^	Extremely preterm-born dropouts n = 7	*p*^2^
Boys	35 (62%)	19 (51%)	0.29	4 (57%)	1.0
Age mean (SD, month)	6y 6mo (4)	6y 7mo (3)		–	–
Gestational weeks, mean (SD)	26^+2^ (1^+6^)	40^+2^(6^+0^)	<0.001^b^	26^+5^ (2^+4^)	0.93^b^
Birth weight, mean (SD), g	822 (278)	3,570 (422)	<0.001	920 (180)	0.38
Small for gestational age	8 (14%)	0	0.02	0	0.29
Mother smoked during pregnancy	6 (11%)	2 (5%)	0.47	3 (43%)	0.02
**Neonatal morbidity**					
Respiratory distress syndrome	43 (77%)	–	–	3 (43%)	0.59
Bronchopulmonary dysplasia at 36^+0^ gestational week	27 (51%)		–	1 (16%)	0.11
Necrotizing enterocolitis	3 (5%)	–		1 (14%)	0.36
Patent ductus arteriosus	44 (79%)	–	–	5 (71%)	0.67
Retinopathy of prematurity*	15 (27%)	–	–	1 (14%)	0.46
Verified sepsis	21 (38%)			2 (29%)	0.65
**Intraventricular hemorrhage**		–	–		0.80
No	36 (64%)	–	–	5 (71%)	
Grade I	5 (9%)	–	–	1 (14%)	
Grade II	9 (16%)	–	–	0	
Grade III	3 (5.5%)	–	–	0	
Grade IV	3 (5.5%)	–	–	1 (14%)	
**White matter injury in MRI at term age**		–	–		0.62
Normal	33 (64%)	–	–	3 (75%)	
Mild	17 (33%)	–	–	1 (25%)	
Moderate	2 (4%)	–	–	0	
Severe	0		–	0	
Mother’s education*			0.005		0.25
High school or lower	26 (46%)	7 (19%)		0	
Bachelor	18 (32%)	11 (30%)		1 (50%)	
Master or higher	12 (22%)	19 (51%)		1 (50%)	
Father’s education*			0.030		NA
High school or lower	29 (54%)	12 (32%)		0	
Bachelor	17 (32%)	11 (30%)		0	
Master or higher	8 (15%)	14 (38%)		1(100%)	
**Cognitive development**					
Full-scale IQ, median (IQR)*	97 (15)	106 (15)	0.001		
Performance IQ, median (IQR)	91 (12)	102 (19)	0.001		
Verbal IQ, median (IQR)*	105 (19)	113 (10)	0.038		

Data are n (%) unless otherwise indicated. p^1^ comparison of the participating extremely preterm-born children (EPT) and term-born controls: p^2^ comparison of the participating and drop-out EPT. IQ, Intelligence quotient; IQR, interquartile range; MRI, magnetic resonance imaging; SD, standard deviation. *Data unavailable: Bronchopulmonary dysplasia (3 EPT and 1 drop-out EPT), Retinopathy of prematurity (1 EPT), White matter injury in MRI (4 EPT and 3 drop-out EPT); Mother’s education (5 drop-out EPT); Father’s education (2 participating EPT and 6 drop-outs EPT) and cognitive development (4 EPT and 3 control children).

### Neurodevelopmental assessments

Neurological examination was performed with Touwen’s comprehensive age-specific qualitative assessment method (Touwen, 1979). The examination consists of eight functional domains: posture and muscle tone, reflexes, involuntary movements, coordination and balance, fine manipulation, associated movements, sensory function, and cranial nerve function (Touwen, 1979; Hadders-Algra, 2010). Dysfunction in one or two domains represents simple minor neurological dysfunction (MND), and in more than two domains, complex MND. In this study, both simple and complex MND were classified as impairment. The Touwen examination shows good reliability for classifications of normal neurological condition, simple MND, and complex MND (*κ* = 0.71–0.83) (Hadders-Algra, 2010).

Motor competence was assessed with the Movement Assessment Battery for Children-2nd edition (MABC-2) manual dexterity, aiming and catching, and balance subtests. A test total score below the 16^th^ percentile (standard score ≤7) was used as a cut-off point for risk of impairment (Henderson et al., 2007). MABC-2 has good to excellent interrater reliability, excellent test–retest reliability, and fair to good validity (Blank et al., 2019).

Sensory integration functions were assessed with the Sensory Integration and Praxis Test (SIPT), applying six independent tests: manual form perception (measures haptic perception and visualization abilities); finger identification (measures tactile perception); design copying (measures visual-motor and visuopraxis); motor accuracy (measures visual-motor coordination); postural praxis (measures the use of proprioceptive information for motor planning), and bilateral motor coordination (measures functional integration of the two sides of the body; Ayres, 1989; Lönnberg et al., 2018). SIPT test scores are reported as z-scores potentially ranging from −3.0 to +3.0 SD (Ayres, 1989). The test raw scores are converted to z-scores [mean = 0, standard deviation (SD) = 1] with a computerized scoring system (WPS, 1996) that uses a normative sample of 1997 US children’s data. The scores +3.0 to −1.0 indicate advanced to typical functioning, −1.0 to −2.0 mild dysfunction, and −2.0 to −3.0 severe dysfunction. The SIPT has very high inter-rater reliability (0.94 to 0.99), and each test has a significant ability (*p* < 0.01) to discriminate between normal and dysfunctional children (Ayres, 1989).

The neuropsychological function was assessed with the Developmental Neuropsychological Assessment, II edition (NEPSY-II), applying four independent domains: attention and executive functioning (auditory attention, visual attention), memory and learning (memory for faces, memory for designs, and narrative memory), sensorimotor functioning (imitating hand positions), and visuospatial processing (arrows, block construction, design copying, and geometric puzzles). The standard scores of NEPSY-II range between one and 19, with a mean (SD) of 10 (3). A test score equal to or less than -1SD (standard score ≤7) was considered as impaired (Korkman and Kemp, 2008). NEPSY-II has adequate to high reliability and good validity in discriminating between different disability conditions (Davis and Matthews, 2010).

General cognitive development, used as background information, was evaluated with the Finnish edition of the Wechsler Preschool and Primary Scale of Intelligence –Third Edition (WPPSI–III) or with the Wechsler Intelligence Scale for Children – Fourth Edition (WISC–IV). Three subtests for Performance Intelligence Quotient (PIQ) (block design, matrix reasoning, and picture completion) and two for Verbal IQ (VIQ) (information and vocabulary) formed Full-Scale IQ (FSIQ) with a mean (SD) of 100 (15) (Wechsler et al., 2009; Wechsler, 2010). The internal consistency of the WPPSI is excellent for both subtests (0.83–0.95) and composite scores (0.89–0.95) and interrater reliability (0.92–0.99). The validity is supported in differentiating gifted children, children with autism, and mental retardation but not children with ADHD (Gordon, 2004). The WISC-IV internal consistency reliability is moderate (0.80–0.89) for subtests and high (>0.90) for overall scores (FSIQ), the criterion validity is highly satisfactory, but the distinguishing validity of various disability conditions is not supported (Andrikopoulos, 2021).

WPPSI-III mean age (SD) at assessment was 6.5y (0.1) and WISC-IV 6.3y (0.2). Three EPT children had recently undergone WISC-IV as part of their clinical follow-up at the hospital due to their low gestational age at birth. Therefore, during their study period, these children could not be reassessed with a similar test, such as the WPPSI-III.

The Touwen neurological examination and MABC-2 were conducted by two experienced child neurologists (authors AL, PL), and the SIPT assessment was by a SIPT-certified (Ayres, 1989) occupational therapist (author UN). The general cognitive development and NEPSY-II were assessed by experienced neuropsychologists (author EW or acquired from hospital records and test record forms).

### Relationship maps of co-occurring impairments

To visualize the co-occurring impairments, we constructed relationship maps with the following 20 test domains: MND (pooled simple and complex MND as a reference for neurological status); MABC-2 manual dexterity, aiming and catching, balance; SIPT manual form perception, finger identification, design copying, motor accuracy, postural praxis, bilateral motor coordination; NEPSY-II auditory attention, visual attention, memory for faces, memory for designs, narrative memory, imitating hand positions, arrows, block construction, design copying, and geometric puzzles. Performance below 1SD of test norms was defined as impaired. For visual clarity, the relationship maps were displayed with three different thresholds: (1) at least one child with the co-occurrence, allowing the visualization of all co-occurring impaired test pairs; (2) at least five children with a co-occurrence, which was the maximum level of co-occurring impaired test pairs in the term-born group; (3) at least ten children with a co-occurrence, allowing visualization of the most frequent co-occurring impaired test pairs in the EPT group.

### Statistical analyses

Data analyses were performed with IBM SPSS Statistics version 28 (IBM Corporation, US) and Matlab version R2020b (Matworks Inc., US). Continuous variables were analyzed with the Mann–Whitney U or *t*-test and reported with median (interquartile range, IQR) or mean (SD). Normality was evaluated with the Shapiro–Wilk test and visually with histograms; homogeneity with Levene’s test and visually with residual plots. The significance level was considered at *p =* 0.05 (2-tailed) or *q* = 0.05 when corrected with a false discovery rate for multiple testing. The confidence interval (CI) was set at 95%. To account for skewed and heteroscedastic data, all continuous data were further analyzed and validated with a bootstrapped *t*-test (*N* samples =1,000) and wild bootstrapped regression analyses (*N* samples = 2000), and bias-corrected and accelerated confidence interval (BCa) at 95% (Gignac, 2019). Multiple regression analyses were adjusted for sex, mother’s education, neonatal brain injury (intraventricular hemorrhage grade III-IV), and a combined neonatal morbidity factor variable (verified sepsis and/or bronchopulmonary dysplasia and/or necrotizing enterocolitis). Cohen’s *d* values were calculated for small (*d* = 0.20), medium (*d* = 0.50) and large (*d* = 0.80) effect size.

Categorical variables were analyzed with *x*^2^, Fisher’s exact or Likelihood ratio, and reported with *n* (%). Odds ratios (OR) for dysfunction were calculated with binomial logistic regression for dichotomous variables and cumulative logistic regression for three-category variables.

Non-systematic missing data in the WPPSI–III were imputed for one EPT in vocabulary, for one EPT in picture completion, and in the WISC–IV for one EPT in vocabulary and picture completion by the mean of the available individual Performance or Verbal subtest score. Non-systematic missing skewed data were imputed with the group median for one EPT in SIPT finger identification and one EPT in SIPT postural praxis and bilateral motor coordination.

The frequency of impaired performance in test domains for each child was plotted for both groups with a bar chart profile. Impairments were further visualized by employing relationship maps, where each test domain was depicted as a node and annotated with the number of children with impaired scores in that domain. The thickness of the line between two nodes was marked to represent the number of children who simultaneously scored poorly (cut off-1SD) in those two domains.

## Results

The EPT and EPT drop-out children did not differ significantly, except that the mothers of the EPT drop-outs were more likely to have smoked during pregnancy than the mothers of the participating EPT children. Compared to the term-born children, the EPT participants were more often boys, and their parents had lower levels of education than the controls’ parents (Table 1). The mothers of the control children recruited at birth had lower levels of education than the mothers of the controls recruited at 6 years. However, the term-born control children recruited at birth and the control children recruited at 6 years did not significantly differ in their test performances.

### Performance in neurodevelopmental assessments

Table 2 reports rates of impairments on the Touwen neurological examination, MABC-2, SIPT, and NEPSY-II tests. EPT children had five times more frequent MND compared to term-born children. The coordination and balance domain was impaired in half of the EPT children but only in 6% of the term-born controls (*q* = 0.004). Almost one-fifth (18%) of the EPT children had the MABC-2 total score below the 16^th^ percentile compared to 3% of the term-born controls (*q* = 0.10). The SIPT finger identification was most often impaired for both groups, in 49% of the EPT and 30% of the term-born children (*q* = 0.13). While the NEPSY-II design copying subtest was difficult for both groups, the EPT children scored below the expected level significantly more frequently (67%) than the term-born children (35%) (*q* = 0.04). Almost half (43%) of the EPT children and one-fifth (21%) of the term-born controls had impairments in NEPSY-II sensorimotor imitation of hand positions (*q* = 0.09).

**Table 2:** Rates of impairments in the Touwen neurological examination, MABC-2, SIPT and NEPSY-II.

Assessment	Extremely preterm-born n/N (%)	Term-born n/N (%)	Odds Ratio, unadjusted (CI 95%)	*q*
**Touwen examination**				
MND*	36/56 (64%)	10/36 (28%)	4.7 (1.7, 11.8)	0.013
Simple MND	22/56 (39%)	9/36 (25%)		
Complex MND	14/56 (25%)	1/36 (3%)		
Posture and muscle tone	22/52 (42%)	8/36 (22%)	2.6 (0.98, 6.70)	0.10
Reflexes	9/52 (17%)	2/36 (6%)	3.2 (0.7, 17.6)	0.16
Involuntary movements	1/52 (2%)	1/36 (3%)	0.7 (0.04, 11.3)	0.79
Coordination and balance	28/52 (54%)	2/36 (6%)	19.8 (4.3, 91.3)	0.004
Fine manipulation	6/52 (12%)	1/36 (3%)	4.6 (0.5, 39.7)	0.19
Associated movements	7/52 (14%)	1/36 (3%)	5.4 (0.6, 46.3)	0.15
Sensory function	5/52 (10%)	0/36 (0%)	NA	0.12^b^
Cranial nerve function	11/52 (21%)	1/36 (3%)	9.4 (1.2 to 76.4)	0.07
**MABC-2, <16**^th^ **percentile**				
MABC-2 Total	9/50 (18%)	1/36 (3%)	7.7 (0.9, 63.7)	0.10
Manual dexterity	13/50 (26%)	1/36 (3%)	12.3 (1.5, 99.0)	0.06
Aiming and catching	3/50 (6%)	1/36 (3%)	2.2 (0.2, 22.4)	0.53
Balance	6/50 (12%)	0/36 (0%)	NA	0.09^a^
**SIPT, <1 standard deviation**				
Manual form perception	10/49 (20%)	0/33 (0%)	NA	0.05^a^
Finger identification	24/49 (49%)	10/33 (30%)	2.2 (0.9, 5.6)	0.13
Design copying	14/49 (29%)	1/33 (3%)	12.8 (1.6, 102.9)	0.07
Motor accuracy	15/49 (31%)	1/33 (3%)	14.2 (1.8, 113.1)	0.06
Postural praxis	16/49 (33%)	4/33 (12%)	3.5 (1.06, 11.7)	0.08
Bilateral motor coordination	19/49 (39%)	3/33 (9%)	6.3 (1.7, 23.7)	0.03
**NEPSY-II, ≤7 scale score**				
*Attention / executive function*				
Auditory attention	12/46 (26%)	1/33 (3%)	11.3 (1.4, 91.9)	0.07
Visual attention	6/45 (13%)	1/34 (3%)	5.1 (0.58, 44.4)	0.17
**Memory and learning**				
Memory for designs	16/45 (36%)	7/34 (21%)	2.2 (0.8, 6.0)	0.17
Memory for faces	9/44 (20%)	5/34 (15%)	1.5 (0.4, 4.9)	0.53
Narrative memory	12/48 (25%)	2/34 (6%)	5.3 (1.1, 25.7)	0.10
**Sensorimotor function**				
Imitating hand positions	19/44 (43%)	7/34 (21%)	2.9 (1.1, 8.2)	0.09
**Visuospatial processing**				
Arrows	8/47 (17%)	1/34 (3%)	6.8 (0.8, 60.0)	0.11
Block construction	9/46 (20%)	1/34 (3%)	8.0 (0.96, 66.8)	0.10
Design copying	33/49 (67%)	12/34 (35%)	3.8 (1.5, 9.5)	0.04
Geometric puzzles	10/45 (22%)	2/34 (5%)	4.5 (0.9, 22.5)	0.10

Comparison with binary logistic regression unless otherwise indicated; *MND, minor neurological dysfunction (simple or complex); ^a^Fisher’s exact; CI, confidence interval; NA, not applicable; OR, the odds ratio for dysfunction in the extremely preterm group; significance level (corrected with false discovery rate), two-tailed q = 0.05. Assessments: MABC-2, Movement Assessment Battery for Children – Second Edition; NEPSY-II, a Developmental Neuropsychological Assessment – Second Edition; Touwen examination, Touwen examination of Minor Neurological Dysfunction; SIPT, Sensory Integration and Praxis Test.

The EPT children had significantly inferior performance to the term-born children across all assessments with continuous variables in *t*-tests, bootstrapped *t*-tests (Supplementary Table 1), and in unadjusted wild bootstrapped regression analyses, except for SIPT bilateral motor coordination and NEPSY-II memory for designs, which were marginally significant (Table 3). When adjusting for sex in multiple wild bootstrapped regression, the group difference was rendered non-significant in MABC-2 aiming and catching. After adjusting for sex, mother’s education, neonatal brain injury, and neonatal morbidity factors, significant differences remained in MABC-2 total score (*q* = 0.01), manual dexterity (*q* = 0.006), balance (*q* = 0.01), and NEPSY-II imitation of hand positions (*q* = 0.008) (Table 3, Model 1).

**Table 3:** Group comparisons of extremely preterm and term-born children in assessments with wild bootstrapped multiple regression.

Assessment	Effect size	Unadjusted			Model 1. Adjusted for sex, mother’s education, neonatal brain injury, and neonatal morbidity factors*			
	*d*	df(x,y)	B (95% BCa)	*q*	df(x,y)	B (95% BCa)	*q*	Adj *r*^2^
**Touwen examination**								
MND			−1.64 (−2.53, −0.75)**	0.001		1.34 (0.12, 2.56)**	0.12	0.28, 0.33***
**MABC-2 (standard score)**								
MABC-2 Total	1.16	(1,84)	−3.29 (−4.77, −1.74)	<0.001	(5,80)	−2.89 (−4.46, −1.14)	0.01	0.40
Manual dexterity	1.31	(1,84)	−3.29 (−4.32, −2.19)	<0.001	(5,80)	−3.20 (−4.54, −1.14)	0.006	0.37
Aiming and catching	0.54	(1,84)	−1.52 (−2.77, −0.13)	0.020	(5,80)	−090 (−2.60, 0.87)	0.41	0.08
Balance	0.90	(1,84)	−2.46 (−3.65, −1.27)	0.002	(5,80)	−2.09 (−3.36, −0.69)	0.014	0.37
**SIPT (Z-score)**								
Manual form perception	0.64	(1,80)	−0.67 (−1.13, −0.21)	0.005	(5,76)	−0.43 (−1.09, 0.25)	0.37	0.07
Finger identification	0.49	(1,80)	−0.58 (−1.04, −0.18)	0.039	(5,76)	−0.60 (−1.32, −0.29)	0.18	0.12
Design copying	1.02	(1,80)	−1.03 (−1.46, −0.59)	<0.001	(5,76)	−0.60 (−1.26, −0.34)	0.16	0.27
Motor accuracy	1.07	(1,80)	−0.94 (−1.26, −0.57)	0.002	(5,76)	−0.65 (−1.26, −0.05)	0.12	0.21
Postural praxis	1.03	(1,80)	−1.01 (−1.41, −0.59)	0.002	(5,76)	−0.68 (−1.30, −0.02)	0.12	0.21
Bilateral motor coordination	0.43	(1,80)	−0.41 (−0.30, 0.16)	0.050	(5,76)	−0.24 (−0.82, 0.44)	0.605	0
NEPSY-II (standard score)								
**Attention/ Executive function**								
Auditory attention	1.10	(1,77)	−2.28 (−3.20, −1.31)	0.001	(5,73)	−1.28 (−2.60, −0.15)	0.13	0.25
Visual attention	0.66	(1,77)	−1.22 (−2.05, −0.33)	0.007	(5,73)	−1.26 (−2.42, −0.13)	0.11	0.10
**Memory and learning**								
Memory for designs	0.44	(1,77)	−1.21 (−2.39, −0.18)	0.049	(5,73)	−0.62 (−2.24, 1.02)	0.55	0.10
Memory for faces	0.75	(1,76)	−1.96 (−3.22, −0.55)	0.006	(5,72)	−1.52 (−2.97, −0.51)	0.14	0.16
Narrative memory	0.80	(1,80)	−2.25 (−3.44, −1.02)	0.007	(5,76)	−1.59 (−3.43, −0.17)	0.15	0.15
**Sensorimotor function**								
Imitating hand positions	0.93	(1,76)	−2.59 (−3.87, −1.32)	0.001	(5,72)	−3.20 (−5.01, −1.18)	0.008	0.22
**Visuospatial processing**								
Arrows	1.12	(1,79)	−2.30 (−3.38, −1.08)	0.001	(5,75)	−1.33 (−2.82, −0.41)	0.14	0.28
Block construction	0.48	(1,78)	−1.38 (−2.66, 0.43)	0.03	(5,74)	−1.28 (−2.94, 0.55)	0.15	0.08
Design copying	0.68	(1,81)	−1.45 (−2.36, −0.38)	0.007	(5,77)	−0.96 (−1.92, 0.14)	0.13	0.07
Geometric puzzles	0.48	(1,77)	−1.30 (−2.44, −0.06)	0.04	(5,73)	−0.74 (−2.61, 1.02)	0.57	0.03

Wild bootstrapped multiple regression (N = 2000): B indicates mean difference in test scores between extremely preterm and term-born participants; 95% BCa, 95% Bias corrected and accelerated confidence interval; Significance level (corrected with false discovery rate), two-tailed q = 0.05; df (x,y), degrees of freedom (regression, residual); Effect size according to Cohen’s d, 0.20 small, 0.50 medium, and 0.80 large; Adj r^2^, variance explained by the adjusted model; IQ, Intelligence quotient; *neonatal brain injury (intraventricular hemorrhage grade III–IV); neonatal morbidity factors (verified sepsis and/or bronchopulmonary dysplasia and/or necrotizing enterocolitis); **Cumulative logistic regression: B indicates unstandardized regression weight with a 95% Wald confidence interval; 3 categories: optimal, simple, and complex Minor Neurological Dysfunction; *** pseudo r^2^, Cox & Nagelkerke; MABC-2, Movement Assessment Battery for Children - Second Edition; NEPSY-II, a Developmental Neuropsychological Assessment - Second Edition; SIPT, Sensory Integration and Praxis Test; Touwen examination, Touwen neurological examination of minor neurological dysfunction.

### Co-occurring neurodevelopmental impairments and relationship maps

**Figure 2:**
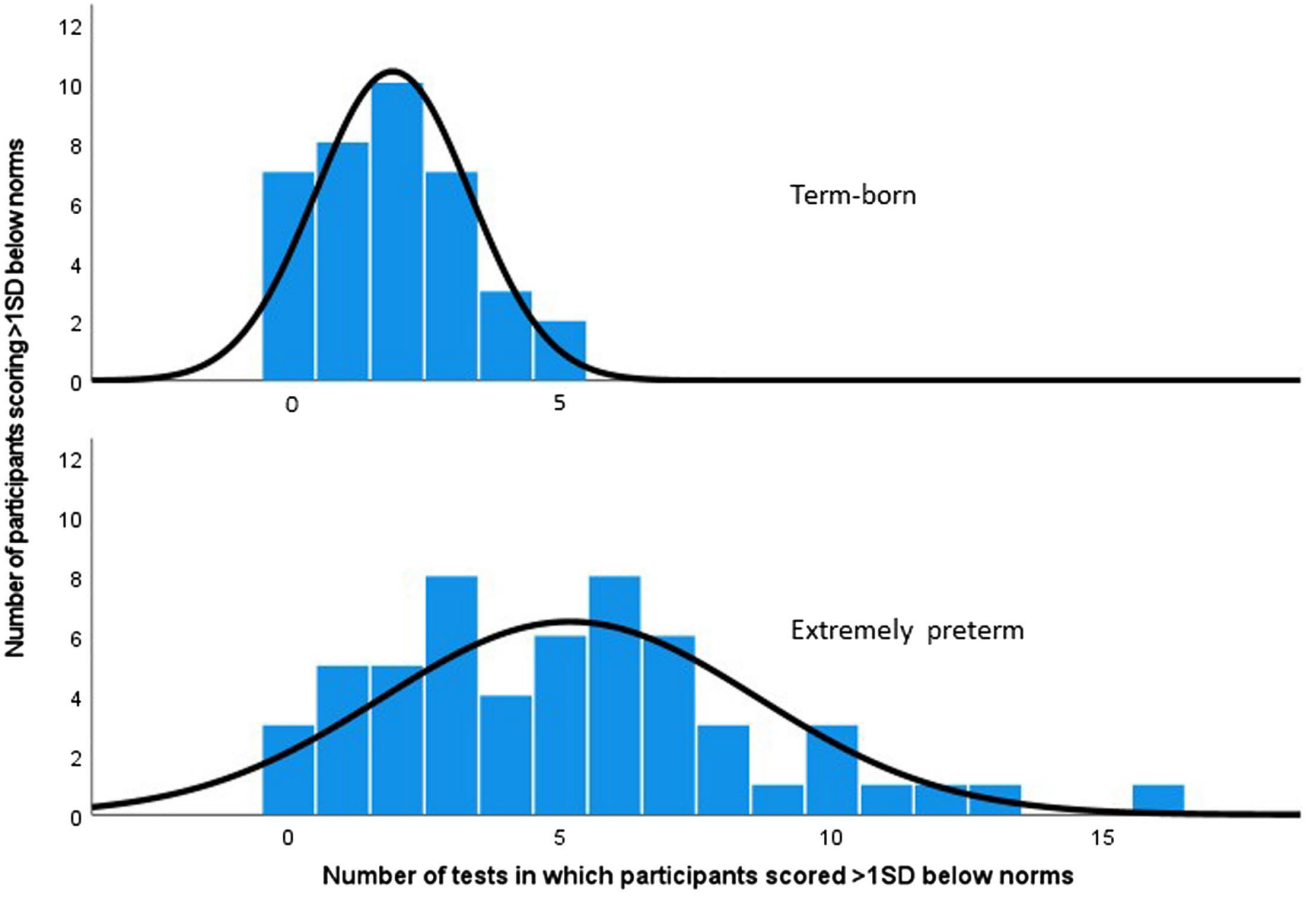
Number of children versus the number of subtests in which 56 extremely preterm and 37 term-born children performed over one standard deviation (SD) below test norms in 20 domains of four assessments. Likelihood Ratio (df) 37.09 (14), *p* < 0.001.

The EPT children had a wider variance in performance and more co-occurring impairments compared to the term-born control children (Figure 2). The mean number of impaired tests was five in the EPT group and two in the term-born group. The maximum number of test domains in which the children scored below expected norms was 16/20 in the EPT and 5/20 in the term-born group (*p* < 0.001). Of the EPT children, 25 (45%) had worse performance than any term-born child (6–16 impaired domains), and 31 (55%) had similar performance to the term-born group (0–5 impaired domains), likelihood ratio, *p* < 0.001.

**Figure 3:**
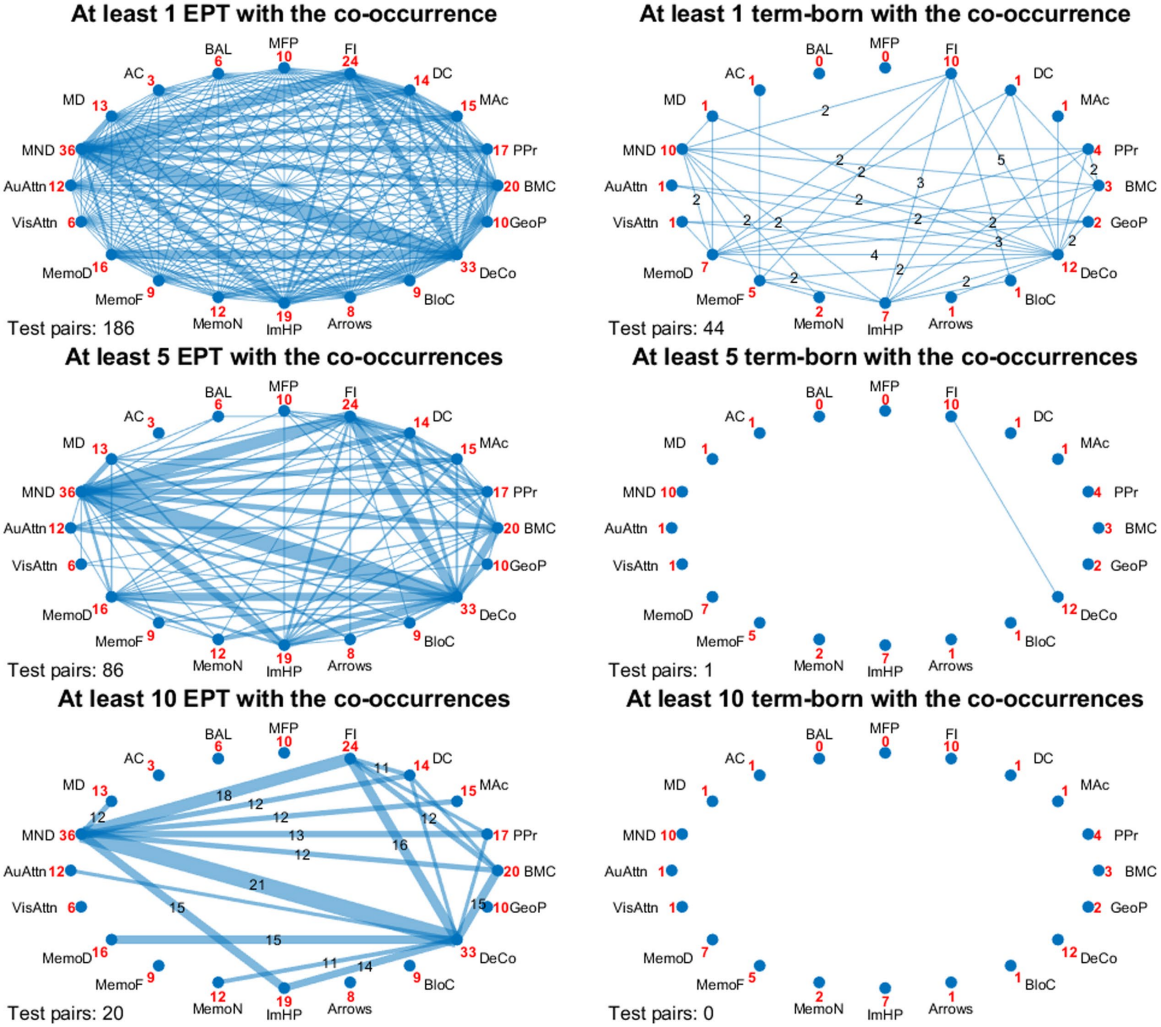
Relationship maps of 56 extremely preterm (EPT) and 37 term-born children who scored over 1SD below test norms concurrently in two test domains (test pairs). Relationships are presented with different cut-offs of 1, 5, and 10 children with co-occurrences. The number (in red) between a test abbreviation and a node indicates the number of children who gave an impaired score in that test domain. The number (in black, when present) above a connecting line indicates children with co-occurring impaired scores in the two line-connected test domains. The number bottom left of each map indicates the total number of impaired test pairs. Test abbreviations are clockwise in the upper semi-circle and anti-clockwise in the lower semi-circle: Upper semi-circle: *Touwen Examination of Minor Neurological Dysfunction*: MND, *Movement Assessment for Children-second edition*: MD, manual dexterity; AC, aiming and catching; BAL, balance. *Sensory Integration and Praxis Test*: MFP, manual form perception; FI, finger identification; DC, design copying; MAc, motor accuracy; PPr, postural praxis; BMC, bilateral motor coordination. Lower semi-circle: *A Developmental Neuropsychological Assessment-second edition*: AuAttn, auditory attention; VisAttn, visual attention; MemoD, memory for design; MemoF, memory for faces; MemoN, narrative memory; ImHP, imitating hand positions; Arrows; BloC, block construction; DeCo, design copy; GeoP, geometric puzzles.

The relationship maps (Figure 3) of the EPT and term-born control children visualize the impaired test performances below 1SD of the test norms. The cut-off point “at least 1 child with the co-occurrence” in Figure 3 indicates that the EPT children had four times more impaired test pairs than the term-born controls (186 vs. 44 impaired test pairs). The second cut-off point (at least 5 children with the co-occurrences) illustrates that the term-born group had a maximum number of five children (5/37) with a co-occurrence, which was in NEPSY-II design copying and SIPT finger identification. The third cut-off point (at least 10 children with the co-occurrences) highlights the 20 most common impaired test pairs in the EPT group. The EPT group had a maximum number of 21/56 children with a co-occurrence in MND and NEPSY-II design copying.

Relationship maps indicate a partly similar tendency in co-occurring neurological, sensorimotor, and neuropsychological impairments in both groups. MND, NEPSY-II design copying, and SIPT finger identification constituted the most prominent relationship of co-occurring impairments in both groups, observed in 33% of the EPT children and 10% of the term-born control children with MND. However, this relationship was ten times more likely in the whole EPT group than in the term-born control group (OR = 10.4, 95% CI [1.29, 84.3], *p* = 0.009). Furthermore, the test performances in NEPSY-II design copying and NEPSY-II memory for design were concurrently below expected norms in 45% of the EPT children with impaired design copying compared to 33% of term-born control children (*p* = 0.11). NEPSY-II design copying and SIPT bilateral motor coordination were concurrently below expected in 36% of the EPT children with impaired design copying and 3% of the term-born children (*p* = 0.002), respectively.

Interestingly, a relationship between MND, NEPSY-II design copying, and NEPSY-II imitation of hand positions was present in the EPT group only, observed in 28% of the EPT children with MND (*p* = 0.004).

## Discussion

This study showed that the EPT children were more likely to perform inferiorly to the term-born children in all neurological, sensorimotor, and neuropsychological test domains. The EPT children had a wider performance variance and considerably more co-occurring impairments than the control children. Nonetheless, over half of the EPT children performed similarly to the term-born group. Both groups also presented a relationship of impairments in MND, NEPSY-II design copying, and SIPT finger identification; however, this relationship was observed more frequently in the EPT group. A relationship between impaired MND, NEPSY-II design copying, and NEPSY-II imitation of hand positions existed only in the EPT group. To our knowledge, this is the first study to report on minor neurodevelopmental impairments using relationship maps and allowing visualization of multiple co-occurring impairments at a glance.

Our study observed MND in 64% of the EPT children and 28% of the term-born controls. Earlier studies have documented MND rates of 36–52% for EPT and 2–23% for term-born children (Broström et al., 2018; Tommiska et al., 2020). Broström et al. applied a simplified Touwen examination with four domains instead of eight, and Tommiska et al. the original Touwen examination with six domains, which may have resulted in a lower prevalence than in our study.

Motor impairments, observed in 18% of the EPT and 3% in the term-born children with MABC-2 in our study were less frequent than previously documented 30–37% (Brown et al., 2015; Bolk et al., 2018a) and 5.5% (Bolk et al., 2018a), respectively. One explanation for these discrepancies might be that the children in our study represented the highest end of the MABC-2 age norms. Accordingly, the tasks might have been too easy to distinguish motor impairments in our children. This explanation is supported by the fact that the term-born children also scored better than expected (Bolk et al., 2018a). Another explanation for our low MABC-2 impairment rates might be that 21 of the EPT children had attended physiotherapy. Of those, 14 still presented MND in the Touwen qualitative examination, but only three showed motor impairments in the MABC-2. This might suggest that while early physiotherapy may have improved motor skills or development, qualitative signs of neurological dysfunction persisted.

Previous EPT studies have reported associations with motor and cognitive impairments (Marlow et al., 2007; Woodward et al., 2009; Broström et al., 2018; Bolk et al., 2018a). Our study found no co-occurrences between motor impairments, measured with MABC-2, and neuropsychological or attentional impairments, presumably due to the low number of motor impairments. Both groups showed co-occurring impairments in NEPSY-II design copying and NEPSY-II memory for designs, which was, however, more apparent in the EPT children compared to the term-born children. Although there were no associations between MABC-2 manual dexterity and cognition, fine-motor impairments became evident in NEPSY-II design copying, which requires fine-motor coordination, visual-motor integration, and also visual working memory, as does the NEPSY-II memory for designs (Ayres, 1989; Korkman and Kemp, 2008). Impairments in fine-motor skills and working memory in EPT children are in line with the results in the previous studies (Marlow et al., 2007; Goyen et al., 2011; Brydges et al., 2018; Bolk et al., 2018b; Doyle et al., 2021).

In the absence of similar studies with relationship mapping, direct comparisons are not possible. However, the results of our study are in line with those of van Hoorn et al. (2010), which found a correlation between poor handwriting, impaired visual-motor integration, and the presence of MND in a study of 8–13 year–old children attending a mainstream school (Van Hoorn et al., 2010). Our study showed impaired NEPSY design copying and SIPT finger identification in the presence of MND in both groups; however, this was ten times more likely in the EPT children compared to term-born controls. While design copying developmentally precedes handwriting (Feder and Majnemer, 2007), NEPSY-II design copying may be considered comparable to handwriting abilities. Both design copying and handwriting require visual-motor integration, perception of directions, hand coordination, and proprioception (Feder and Majnemer, 2007). In addition, both require a functioning pencil grasp through awareness of fingers and accurate tactile processing. In our study, tactile processing measured by SIPT finger identification (Ayres, 1989) was challenging for both groups.

Interestingly, the relationship between impaired MND, NEPSY-II design copying, and NEPSY-II imitation of hand positions was present in the EPT group only. The difference between the co-occurrences of SIPT finger identification in both groups and NEPSY-II imitation of hand positions only in the EPT group is intriguing and may be related to task complexity. While the SIPT finger identification requires touch differentiation in one or two fingers without vision, the NEPSY-II imitation of hand positions is more complex. It requires visual-and sensorimotor perception for spatial relationships, motor planning, visual motor imitation abilities, coordinated movements in hands and fingers, and comprehension, among others. (Ayres, 1989; Korkman and Kemp, 2008). Thus, the EPT children with multiple underlying minor impairments were more likely than the controls to face difficulties in complex tasks. Consequently, the accumulation of underlying impairments may render seemingly ordinary tasks, such as imitations of motor activities, difficult for EPT children to perform.

The co-occurring impairments among EPT children may be related to widely distributed disturbances in normal brain development (Kostović and Jovanov-Milošević, 2006). EPT children are susceptible to brain injury, altered brain maturation (Volpe, 2009; Back, 2015), impaired growth of brain structures such as cerebral cortex, basal ganglia, thalamus, cerebellum (Volpe, 2009; de Kieviet et al., 2012; Matthews et al., 2018; Dewey et al., 2019), and microstructural alterations (Groeschel et al., 2014; Kelly et al., 2016) that are linked to impaired motor functions (Groeschel et al., 2014; Biotteau et al., 2016; Kelly et al., 2016; Matthews et al., 2018) and MND (Hadders-Algra, 2002; Setänen et al., 2016). In our cohort, MND and impaired NEPSY-II design copying exhibited the most frequent co-occurrence in impairments among the EPT children. The most prevalent domain contributing to MND was coordination and balance, which has been associated with cerebellar dysfunction (Manto et al., 2012). Furthermore, impaired NEPSY-II design copying that measures visual-motor function, might reflect cerebellar and dorsal-visual pathway disturbances (Fazzi et al., 2004; Van Braeckel and Taylor, 2013). The role of cerebellar and visual pathway disturbances contributing to MND and NEPSY-II design copying impairments remains an option for future research.

The strength of our study is the comprehensive qualitative and quantitative testing of neurological, sensorimotor, and neuropsychological functioning, enabling a view of co-occurring impairments. While the statistical analyses explored the significance of group differences test by test, the relationship maps depicted impairments in all tests and their co-occurrences in both groups. The relationship mapping allowed us to compare specific configurations in both groups.

There are limitations to the study. The relatively small sample size created a risk for type II error and assumption violations in statistical analyses. Therefore, we compared normative analyses to bootstrapped analyses, which supported rejecting the null hypothesis. Our control group was a convenience sample recruited in two periods. As this posed a risk for selection bias, we compared the performance of the control children recruited at birth and control children recruited at 6 years to verify possible differences and found no differences in the test performances. The control group also presented a lower boy/girl ratio and a higher socioeconomic status relative to the EPT group. We, therefore, adjusted for these confounders (sex and mothers’ education) to avoid bias. Finally, the large number of applied tests may have increased the probability of finding impairments. However, evaluating a more limited range of tests might have led to underestimating the extent of multiple impairments in EPT children.

Our study indicated that a significant proportion of EPT children show multiple co-occurring minor impairments at 6 years of age. Thus, the EPT children who are observed clinically to have a single impairment are likely to exhibit multiple co-occurring impairments, which may negatively interfere with their performance and participation in daily, social, and academic activities. When expectations increase with age, multiple minor impairments become more apparent (Woodward et al., 2009; Hutchinson et al., 2013; Johnson et al., 2016; Spittle et al., 2018; Doyle et al., 2021). This implies that young children born EPT need careful monitoring at least until school age for signs of minor co-occurring impairments. Timely, individualized, multidisciplinary intervention methods are needed to support the neurodevelopment of complex abilities. These methods should also consider guidance and support for parents to optimally engage EPT children in daily interactions and activities, which research has shown to support positive neurodevelopment (Rahkonen et al., 2014).

## Data availability statement

The data supporting the conclusions of this article will be made available by the authors on reasonable request.

## Ethics statement

The studies involving human participants were reviewed and approved by The Ethics Committee for gynecology and obstetrics, pediatrics, and psychiatry of the Hospital District of Helsinki and Uusimaa. Written informed consent to participate in this study was provided by the participants’ legal guardian/next of kin and the participant.

## Author contributions

UN, AL, and MM contributed to the study design. UN, AL, PL, and EW contributed to the data acquisition. UN conducted the statistical analyses and drafted the initial manuscript. UN, AL, MM, and PL contributed to interpreting the data and results. UN, AL, MM, PL, and EW contributed to the drafting and revision of the manuscript for important intellectual content. All authors have complete access to the study data, read and approved the final version of the manuscript, and agreed to its submission.

## Funding

The study was supported by Foundation for Pediatric Research (Lastentautien tutkimussäätiö), Arvo and Lea Ylppö Foundation (Arvo ja Lea Ylppö Säätiö), and Pediatric Research Center (Lastentautien tutkimuskeskus). The funding organizations were not involved in the design of the study, data collection or analyses, interpretation, or publication of the results.

## Conflict of interest

The authors declare that the research was conducted in the absence of any commercial or financial relationships that could be construed as a potential conflict of interest.

## Publisher’s note

All claims expressed in this article are solely those of the authors and do not necessarily represent those of their affiliated organizations, or those of the publisher, the editors and the reviewers. Any product that may be evaluated in this article, or claim that may be made by its manufacturer, is not guaranteed or endorsed by the publisher.

## Abbreviations

EPT: Extremely preterm-born
MND: Minor neurological dysfunction
MABC-2: Movement Assessment Battery for Children – Second Edition.
NEPSY-II: A Developmental Neuropsychological Assessment – Second Edition.
SIPT: Sensory Integration and Praxis Test.
