# Supplementary material for: Extremely preterm children and relationships of minor neurodevelopmental impairments at 6 years
Source: Front Psychol. 2022 Dec 1;13:996472. doi: 10.3389/fpsyg.2022.996472 (PMC9751855; doi:10.3389/fpsyg.2022.996472)
Supplement: Supplementary file 1 [file Table_1.pdf]

Supplement table 1. Group comparisons with t-test and bootstrapped t-tests

Assessment	N (EPT, term-born)	Mean difference, (95%CI)	t-test, *q*	95% BCa	Bootstr. t-test, *q*
**Touwen examination**					
MND*			0.002**		0.003**
**MABC-2 (standard score)**					
MABC-2 total	50, 36	3.3 (2.12, 4.60)	<0.001	2.15, 4.64	0.01
Manual dexterity	50, 36	3.3 (2.2, 4.37)	<0.001	2.27, 4.50	0.02
Aiming and catching	50, 36	1.5 (1.27, 3.64)	0.02	0.23, 2.75	0.01
Balance	50, 36	2.5 (1.34, 3.6)	<0.001	1.40, 3.58	0.003
**SIPT (Z-score)**					
Manual form perception	49, 33	0.67 (0.26, 1.09)	0.002	0.28, 1.08	0.01
Finger identification	49, 33	0.57 (0.49, 1.10)	0.040	0.09, 1.04	0.022
Design copying SIPT	49, 33	1.03 (0.61, 1.46)	<0.001	0.64, 1.43	0.003
Motor accuracy	49, 33	0.94 (0.58, 1.30)	<0.001	0.58, 1.29	0.005
Postural praxis	49, 33	1.01 (0.57, 1.45)	<0.001	0.59, 1.43	0.003
Bilateral motor coordination	49, 33	0.41 (-0.01, 0.83)	0.06	0.50, 0.75	0.06
**NEPSY-II (standard score)**					
Attention/ Executive function					
Auditory attention	46, 33	2.28 (1.40, 3.16)	<0.001	1.31, 2-94	0.004
Visual attention	45, 33	1.22 (0.38, 2.05)	0.007	0.31, 2.03	0.020
Memory and learning					
Memory for designs	45, 34	1.21 (-0.23, 2.45)	0.06	-0.03, 2.58	0.03
Memory for faces	45, 34	1.96 (0.78, 3.14)	0.002	0.82, 3.36	0.004
Narrative memory	48, 34	2.25 (0.99, 3.51)	0.001	0.90, 3.50	0.007
Sensorimotor function					
Imitating hand position	44, 34	2.59(1.32, 3.87)	<0.001	1.30, 3.92	0.002

Visuospatial processing					
Arrows	47, 34	2.30 (1.43, 3.18)	<0.001	1.26, 3.23	0.007
Block construction	46, 34	1.38 (0.10, 2.66)	0.04	0.64, 2.67	0.04
Design copying	49, 34	1.45 (0.50, 2.39)	0.005	0.58, 2.58	0.006
Geometric puzzles	45, 34	1.30 (0.61, 2.53)	0.038	0.81, 2.63	0.02

Group comparisons with t-tests; t-test with bootstrapping, N(samples)=1000; BCa, Bias-corrected and accelerated confidence interval; CI, confidence interval for mean difference; EPT, Extremely preterm-born children; significance level (corrected for false discovery rate, 21 comparisons) two-tailed, $q = 0.05$; * MND, Minor neurological dysfunction, combined simple and complex; ** x^2
